# Optimizing breast cancer screening strategies for women with different BMI levels in Ghana: A simulation-based study on BMI-dependent tumor growth model

**DOI:** 10.1371/journal.pgph.0004953

**Published:** 2025-07-28

**Authors:** Asamoah Larbi, Eric Nyarko, Samuel Iddi

**Affiliations:** 1 Department of Statistics and Actuarial Science, School of Physical and Mathematical Sciences, University of Ghana, Legon, Accra, Ghana; 2 African Population and Health Research Center, APHRC Campus, Nairobi, Kenya; Islamic Azad University South Tehran Branch, IRAN, ISLAMIC REPUBLIC OF IRAN

## Abstract

Breast cancer is a disease in which abnormal cells in the breast tissue grow out of control to form tumors and can spread to other parts of the body. While it can affect both men and women, it poses a greater risk to women, and it is a leading cause of cancer-related deaths worldwide. This study aimed to examine different mammography screening interval strategies using a body mass index (BMI)-dependent tumor growth model and a simulation approach. The goal was to identify the optimal screening strategy for various BMI levels by investigating the association between BMI and tumor growth rate, and further examine the relationship between BMI and screening outcomes, using a continuous growth model and Cox regression, respectively. Our results indicated that a biennial screening interval yielded the best outcomes for all BMI levels compared to annual and triennial strategies. Obese individuals may require higher screening sensitivity and are likely to benefit from shorter screening intervals than those with other body weights within the screening age range of 30 to 65 years. Additionally, obese individuals have a slightly higher risk of being diagnosed with interval-detected cancers rather than screen-detected cancers. In contrast, women with a normal body weight have a greater chance of being detected through screening rather than at intervals. These findings suggest that breast cancers may become symptomatic more quickly in obese individuals than in those with lower body weights. Consequently, the standard two-year screening interval may not be optimal for this group, indicating that more frequent screenings (14-18 months) could be necessary. This underscores the potential impact of improved screening practices to enhance the treatment and management of breast cancer.

## Author summary

Breast cancer, a disease that affects both women and men, is a significant global health concern. It is a leading cause of cancer-related deaths worldwide, with women facing a significantly higher risk. In 2020, it accounted for nearly one in eight cancer cases globally, making it the most concerning type of cancer for women. The year saw approximately 2.3 million new cases of breast cancer and around 685,000 deaths. By 2040, these numbers are estimated to rise to about 3 million new cases and 1 million deaths, underscoring the urgent need for effective screening strategies. In this study, we focused on incorporating body mass index into the tumor growth sub-model of the continuous growth model, and used a simulation approach to evaluate different strategies for mammography screening intervals and further examine BMI category association with screening outcomes. Our results showed that biennial screening is the most beneficial approach for all BMI levels, surpassing annual and triennial strategies. Furthermore, it was found that obese individuals, due to their increased need for screening sensitivity, may benefit from more frequent screenings than those with other body weights within the 30 to 65-year screening age range. It is important to note that obese individuals have a slightly higher risk of being diagnosed with interval-detected cancers rather than screen-detected cancers. In contrast, women with normal body weight are more frequently identified during screenings than between screenings. These findings suggest that breast cancers may become symptomatic more quickly in obese individuals than in those with lower body weights, indicating the necessity for more frequent screenings (14-18 months). This underscores the importance of the research findings and their potential implication for screening policies.

## Introduction

Breast cancer is a disease in which abnormal cells in breast tissue grow uncontrollably, forming tumors [1, 2]. These abnormal cells can potentially spread (metastasize) to other body parts if left untreated [1]. Breast cancer can be classified based on various factors, including pathological history, staging, grading, and differentiation systems [3–5]. This disease poses a significant threat, particularly to women, and it is a major contributor to cancer-related deaths worldwide [6]. In 2020, approximately 1 in 8 cancer cases globally were attributed to breast cancer, with around 2.3 million new cases and approximately 685,000 deaths reported [7]. Projections suggest that by 2040, the number of new cases could rise to about 3 million, with deaths increasing to 1 million [8].

Population-based mammography screening programs are crucial for the early detection of breast cancer [9]. However, the future looks promising with ongoing efforts from researchers and stakeholders to improve the effectiveness of these screenings. Policies, including personalized screening programs that adjust recommendations based on individual risk factors, are increasing [10]. Tailoring breast cancer screening to individual risks can significantly reduce the adverse effects of false positives and overdiagnosis. This personalized approach not only improves screening effectiveness and survival rates but also minimizes harm and maximizes the benefits of the programs [11], giving hope for more effective breast cancer detection programs in the future.

For example, empirical evidence highlights the benefits of personalized breast cancer screening programs, although concerns about feasibility and acceptability remain [12]. A study has reported high satisfaction rates with individualized screening intervals based on risk factors [13]. Breast cancer risk factors are attributes that increase the likelihood of developing the disease [14]. These factors can be behavioral, physiological, demographic, genetic, or environmental. Studying these risk factors is essential to understanding the outcomes of breast cancer based on population characteristics. Key risk factors include hereditary elements, such as family history [15], high body weight [16, 17], high breast density [18], hormone replacement therapy (HRT) [19, 20], late marriage and childbirth, age and menopausal status, as well as smoking and alcohol consumption [21]. Exposure to radiation and certain chemicals [22], along with health-related factors such as diabetes [23], cardiovascular health [24], and bone density [25], are also significant. In addition, advanced tumor characteristics, such as locally advanced breast cancer, metastatic breast cancer, and inflammatory breast cancer, are associated with a high body mass index (BMI) [26, 27] and other risk factors, which increase the relevance of personalized diagnosis and treatment for breast cancer. In particular, research has shown that high BMI correlates with clinical diagnoses of advanced-stage breast cancer [28] and lymph node metastasis [28, 29]. This finding sheds light on the significant role of BMI in tumor growth [30, 31], especially in aggressive tumors such as triple-negative breast cancer (TNBC) [32, 33]. However, no association has been found between BMI categories and the prognosis of TNBC [31, 34, 35]. Tumor growth factors, including tumor size, growth rate, and presence time, play an important role [36]. Studies indicate that an average tumor size ranging from 7 mm to 60 mm is associated with axillary lymph node involvement [37], metastases, and mortality rates [38]. A high BMI is associated with larger tumors [28, 29], faster growth, or shorter doubling times [39], highlighting the importance of BMI in understanding and predicting tumor growth.

These empirical findings underscore the potential advantages of personalized screening intervals based on BMI. For example, women with rapid tumor growth may benefit from more frequent screenings before a tumor becomes clinically detectable [40]. However, several factors are crucial in determining the appropriate screening interval, including the preclinical phase of breast cancer (also known as tumor presence time) and tumor doubling time (i.e., the time it takes for a tumor to double in size or volume) [41]. Understanding factors such as BMI, which are linked to tumor growth, is vital to planning and evaluating screening programs [36]. Simulation studies can provide evidence-based guidelines for policymakers, utilizing innovative personalized methods such as incorporating risk factors into continuous growth sub-models [42] and stratifying individual risk using hazard models [11].

Studies contributing to personalized medicine, particularly regarding the influence of risk factors on the natural history of breast cancer, have examined multiple risk factors. Firstly, some studies have evaluated how factors such as BMI, family history, age at marriage and childbirth, age, and menopausal status impact the risk of tumor onset by integrating these factors into tumor onset sub-models [43]. Secondly, the effects of hormone replacement therapy, BMI, and other relevant factors on tumor growth rates have been assessed through tumor growth sub-models [42, 43]. Thirdly, the relationships between breast size, breast area, and BMI concerning the risk of symptomatic detection have been investigated [42, 43]. Lastly, the impact of breast density on screening sensitivity has also been evaluated within the screening sensitivity sub-model [43].

The study objectives, design, and available data have guided the selection of risk factors in past studies [42, 43]. Although many studies have incorporated several risk factors to elucidate the natural history of breast cancer and to inform screening policies [42, 43], little is known about their application in middle- to low-income countries, particularly in sub-Saharan Africa, such as Ghana. This lack of knowledge is largely attributed to the absence of mammography screening programs and relevant screening-specific data. Furthermore, while previous research has explored the connections between risk factors and breast cancer development, as well as their interactions, few studies have aimed to apply these findings at the individual level, which is essential for implementing personalized public health interventions.

Our study sought to address these gaps by examining various mammography screening strategies. We explored different screening intervals—annual (every 12 months), biennial (every 24 months), and triennial (every 36 months)—for women aged 30 to 65 years. We utilized a simulation-based tumor growth model that accounted for BMI. Our objectives were to investigate the relationship between BMI and tumor growth rate (using a continuous BMI variable to capture the complexity of tumor growth) and to analyze the association between BMI and screening outcomes (using categorical BMI variables to examine screening outcomes). These outcomes included cancers detected during scheduled mammography screenings (referred to as screen-detected cancers) and cases identified between screenings (known as interval-detected cancers, occurring after previous negative mammography results). By pursuing these objectives, we aimed to develop optimal screening strategies tailored to various BMI levels and to enhance our understanding of how BMI influences the breast cancer disease process, along with the implications for screening and early detection strategies. We employed continuous growth models to achieve our objectives, which have proven effective for modeling cancer data, including insights into natural history and screening programs [42, 44–47]. Additionally, we used Cox regression analysis to evaluate how BMI influenced screening outcomes, adjusting for the age at detection. This analysis incorporated cause-specific hazards, subdistribution hazard ratios, and cumulative incidence estimates. Due to their reliance on specific data, continuous growth models offer valuable contributions to population-based screening programs [45]. However, to facilitate their application in our study, some parameters were borrowed under careful assumptions, which exposed the study to potential uncertainties. Furthermore, the empirical data (tumor size and BMI) utilized for estimating tumor growth rates and age at symptomatic parameters were derived from clinical data concerning patients diagnosed with invasive breast cancers, including invasive carcinoma, ductal carcinoma, papillary carcinoma, lobular carcinoma, metastatic carcinoma, and infiltrating carcinoma. These cases are often associated with larger tumors and potentially higher BMI individuals [28, 29], which can affect tumor growth rate estimates, as larger tumors tend to be linked with slower growth rates [46].

Additionally, our study could only incorporate BMI into the growth rate sub-model due to limited data availability during the research. To our knowledge, this is the first effort of its kind in the context of Ghana. However, the reliance on a single risk factor restricted our ability to make extensive contributions toward personalized screening. Nonetheless, discussions surrounding personalized screening that targets different BMI categories have gained considerable attention in the research community, particularly due to the poor prognostic outcomes associated with obese patients [48–50]. This makes our study relevant to guide policy design. This effort is critical as it contributes to personalized screening by examining the relationship between BMI and screening outcomes, which can help propose specific schedules tailored to different BMI levels. Our study has the potential to significantly impact the field of oncology by optimizing breast cancer screening programs in middle- and low-income countries, including Ghana, and guiding policy decisions to enhance their effectiveness.

## Continuous growth models

### Tumor onset

In a previous study, [45] adopted the classical Moolgavkar-Venson-Knudson (MVK) two-stage model of carcinogenesis, which hypothesized that cancer may be formed after normal cell experience two mutation events with four Poisson distributed events rates namely cell division rate ($\tilde{\alpha }$), first event rate ($\tilde{\nu }$), cell death rate ($\tilde{\beta }$), and second event rate ($\tilde{\mu }$). Following the four parameters ($\tilde{\alpha },\;\tilde{\nu },\;\tilde{\beta },\;\tilde{\mu }$) identifiability limitation for diagnostic data, [45] modeled the probability of tumor onset at age *t* based on the survival function:

$$\;F_{T}(t)=P(T>t)={\left[\frac{(B-A)e^{Bt}}{Be^{(B-A)t}-A}\right]}^{\delta },$$ (1)

where *A*, *B*, and δ are the model parameters defined in relation to the Poisson distributed mutation events rate as shown in the functions below (Eqs 1.1–1.3). This reparameterization enhanced parameter identifiability [45] and even reduced computational complexity for the disease onset risk simulation.

$$\delta =\tilde{\nu }/\tilde{\alpha },$$ (1.1)

$$A=\frac{1}{2}[(\tilde{\beta }+\tilde{\mu }-\tilde{\alpha })+\sqrt{(\tilde{\beta }+\tilde{\mu }-\tilde{\alpha }{)}^{2}+4\tilde{\alpha }\tilde{\mu }}],$$ (1.2)

$$B=\frac{1}{2}[(\tilde{\beta }+\tilde{\mu }-\tilde{\alpha })-\sqrt{(\tilde{\beta }+\tilde{\mu }-\tilde{\alpha }{)}^{2}+4\tilde{\alpha }\tilde{\mu }}],$$ (1.3)

The model parameters (*A*, *B*, and δ) were borrowed from [47] due to a lack of screening-specific data in Ghana. However, the time at which the risk of disease onset was evaluated *t*, was adjusted to the Ghanaian population using a theoretical assumption based on comparative age-standardized incidence rates for West Africa (Ghana) and Northern Europe (Sweden) according to the GLOBOCAN report on global breast cancer statistics [7]. For example, the age-standardized incidence for Northern Europe was 86.4 compared to 41.5 for West Africa; therefore, we expected that the age *t* at risk evaluation would be 0.48 (41.5/86.4 = 0.48) earlier for West Africa [51]; see the evaluation time *t* in Table 2. Furthermore, to justify the suitability of the estimates borrowed for the model parameters *A*, *B*, and δ for the assumption of the risk of disease onset for Ghana, a sensitivity analysis was carried out by varying the parameter estimates by ±10% and comparing the screening results (See S1 Table). However, despite the careful application of the borrowed parameter estimates to the Ghanaian population, the approach introduces potential limitations into the current study due to genetic variation between Ghanaian and Swedish populations.

**Table 1:** Descriptive summary of data.

Variable	Mean (SD)	Min	1*^st^* Quartile	Median	2*^nd^* Quartile	Max
Tumor size (mm)	51.38 (±30.35)	10.00	29.00	45.00	68.50	130.00
BMI (kg/m^2^)	29.73 (±7.46)	14.96	24.64	28.81	33.84	67.46
Age (years)	51 (±12)	26	41	49	60	85

*^NB^* Numbers of folders with detailed tumor size and BMI reviewed, *n* = 187

**Table 2:** Simulation parameters.

Model	Parameter	Estimate	Method of estimation
Eq (1)	A,B,δ	-0.0722, 1.18x10^−3^ 0.0952	Literature [47]
Eq (1)	*t*	0.48*t ((41.5/86.4)=0.48)	Theoretical assumption [7, 47]
Eq (5)	γ	6.418*x*10^−5^	Empirical data (Tumor size)
Eq (6)	$\beta_{0},\;\beta_{1}$	-5.04, 0.56	Literature [44]

### Tumor growth

Given the age at tumor onset *T* = *t*, and an inverse tumor growth rate *r*, as applied in [45, 47], we similarly estimated tumor volume *V*(*t*) at age x≥t as

$$V(t)=\left\{ \begin{array}{c} \nu_{o}e^{\frac{z}{r}},\;\;t>0\\ 0\;\;otherwise \end{array}\right.,$$ (2)

where *z* is the difference between age at detection (*x*) and age at tumor onset (*t*) (i.e. *z* = *x*−*t*), and we assumed tumor to be spherical with diameter 0.5 mm, such that tumor volume $\nu_{0}\approx {0.065mm}^{3}$; where the time constant inverse growth rate *r* relate to tumor doubling time (DT), DT=ln(2)r, such approach limited computational burden. Again, we accounted for the heterogeneity of different tumor growth by assuming that the inverse growth rate *r* in Eq (2), is a random variable *R*, drawn from a gamma distribution with shape and scale parameters *a*, and *b* respectively as expressed below [47], however because we did not have screening data, we constrained the growth rate parameters such that *a* = *b* for identifiability purpose.

$$f_{R}(r)=\left\{ \begin{array}{c} \frac{b^{a}r^{a-1}e^{-br}}{\Gamma (a)},\;\;a=b,r>0\\ 0\;\;otherwise \end{array}\right..$$ (3)

#### BMI-dependent tumor growth rate.

To account for BMI-dependent tumor growth rate, we integrated BMI into the tumor growth sub-model of the continuous growth models, as proposed by [42] and [45], to elucidate the BMI (continuous variable) on the tumor growth rate. We considered the covariate interaction based on the assumption that the expectation *E* of the inverse tumor growth rate *r*, modeled as a gamma-distributed random variable *R* in Eq (3), can be expressed as $E(R)=\frac{a}{b}=\mu$. This led to the necessity of reparametrizing the mean as follows:


$$Var(R)=\frac{a}{b^{2}}=\left(\frac{1}{a}\right){\left(\frac{a}{b}\right)}^{2}=\sigma^{2}\mu^{2}.$$

Here, $\sigma^{2}=\frac{1}{a}$, where σ is the coefficient of variation, also for the purpose of interpretation let’s refer to $\sigma^{2}$ as ϕ [42, 45].

Now, we allow growth rates to vary with the BMI risk or covariate by incorporating the continuous BMI variable using a log-linear mean function.

$$\log(\mu )=\lambda_{0}+\lambda_{1}x,$$ (4)

where μ is the expectation *E*, of the gamma-distributed random variable *R*, *x* is a continuous variable BMI, $\lambda_{0}$ is an intercept term and $\lambda_{1}$ is the coefficient of the BMI covariate, which is the estimate of a unit change of BMI on the mean of the tumor growth rate. With the help of this estimate, we could account for the BMI of every individual in the simulated population to interpret the screening results and characteristics of the natural history at the individual BMI levels, Here, we use the maximum likelihood estimation approach from previous studies [46, 52] as derived in [51] to estimate the parameters $\lambda_{0}$, $\lambda_{1}$ and ϕ using empirical data, instead of *a* and *b* in Eq 3 [51], the results are shown in Table 3. The tumors used with the BMI were invasive breast cancers, which may affect the growth rate estimates, as larger tumors tend to grow slowly.

**Table 3:** Estimates of tumor growth rate with covariate.

Parameter	Point estimation	Bootstrap 95% CI
$\lambda_{0}$	-0.015	(-0.215, 0.224)
$\lambda_{1}$	0.009	(-0.001, 0.017)
ϕ	1.355	(1.107, 1.588)

### Symptomatic detection

The rate at which symptoms are noticed by individuals at time *t* based on [45] assumptions is given as:

$$P(T_{Sy}\in (t,t+\Delta t]|T_{Sy}>t)=\gamma V(t)\Delta t+o(\Delta t),\;\gamma >0,$$ (5)

where γ measures hazard rate, the parameter was estimated using empirical data as shown in Table 2; however, since the data is from a single prominent facility and involves clinical cases, the estimate may not reflect the average population. *T*_*Sy*_ is the time from onset to symptomatic detection, which is assumed to follow a continuous hazard rate function proportional to the current (latent) tumor volume *V*(*t*), and Δt is a small change in time. Given that age at symptomatic detection is denoted as *U*, then let time from onset to symptomatic $T_{Sy}=U^{\prime }$, such that $U=T\;+U^{\prime }$, where *T* is age at disease onset [45], we calculated tumor volume at symptomatic *V*(*U*), tumor size at symptomatic *d*(*U*), and time to symptomatic detection $U^{\prime }$ using the following functions.

$$V(U)=\nu_{o}-\frac{\log(1-u)}{\eta r}$$ (5.1)

$$d(U)=\sqrt[{3}]{\frac{6}{\pi }V(U)},\;U\geq T,$$ (5.2)

$$U^{\prime }=r\ln\left(\frac{V(U)}{\nu_{o}}\right),$$ (5.3)

where *u* in Eq 5.1 is uniform distribution and *r* is the inverse tumor growth rate (See Eq 3).

### Screen detection (Screening sensitivity)

Assuming that every woman will undergo a mammography screening at a specific age, denoted as *x*, there are two possible outcomes: a tumor may be detected or not. The probability of detecting an existing tumor during the screening at age *x* is expressed as a function of tumor size (or diameter) *d*(*x*). It is assumed that larger tumors are more likely to be detected more quickly. This relationship is represented by the probability of detecting a tumor based on its diameter at age *x*, *P*_*d*(*x*)_[45]

$$P_{d(x)}=\left\{ \begin{array}{c} \frac{e^{\beta_{o}+\beta_{1}d(x)}}{1+e^{\beta_{o}+\beta_{1}d(x)}},\;x>T,\\ 0\;\;otherwise \end{array}\right.,$$ (6)

where $\beta_{0}$ and $\beta_{1}$ are sensitivity parameters, these parameters were borrowed from [44] and calibrated to an early screening age of 30-65 in the Ghanaian population due to the lack of screening data and the sensitivity analysis carried out based on the variation of the estimate borrowed by ±10% (See S2 Table). However, these estimates introduce likely limitations, since the sensitivity could vary for the Ghanaian population due to regional differences. Here *d*(*x*) is the diameter of the tumor at age (*x*):

$$d(x)=\sqrt[{3}]{\frac{6}{\pi }\nu_{o}e^{\left(x-t\right)/r}},\;x\geq t.$$ (6.1)

where $\nu_{0}$ is the tumor volume at onset, *t* is the age at tumor onset, and *x* is the age at tumor detection (See Eq 2); *r* is the inverse tumor growth rate (See Eq 3).

## Method

### Ethics statement

The study was approved by the Scientific and Technical Review Committee of Korle Bu Teaching Hospital, and ethical approval was granted by the Institutional Review Board (IRB) of Korle Bu Teaching Hospital (Reference No.: KBTH-IRB 00058/2023). The data were fully anonymized before being accessed, and the IRB ethics committee waived the requirement for informed consent.

### Data description

A total of 187 data related to tumor size and BMI from 2018 to 2022 were collected from breast cancer cases at Korle Bu Teaching Hospital from 5/10/2023 to 3/11/2023. The data for this study are summarized in Table 1. The mean values recorded were as follows: tumor size/diameter measured 51.38 mm (standard deviation (SD): ± 30.35), BMI was $29.73\;{\text{kg/m}}^{2}$ (SD: ± 7.46), and the average age of the patients was 51 years (SD: ± 12).

## Simulation of screening program

### Simulation parameters

In this study, we selected the screening population age range of 30 to 65 based solely on the age distribution of our empirical data outlined in Table 1. Our primary focus was to examine the association rather than the characteristics of the eligible screening population [53, 54] as discussed in our previous work [51]. This distinction is crucial, as the eligible population may be linked to screening harms, such as overdiagnosis [51, 54, 55]. Since our primary interest lies in establishing the association, the characteristics of the eligible population were not a significant concern. Additionally, our analysis focused on body mass index (BMI) as a covariate, given its availability in the dataset and established prognostic relevance. Unfortunately, we were unable to incorporate other essential risk factors, such as family history and hormone replacement therapy, into our sub-models due to a lack of data on these variables. This limitation restricts the extent to which our findings can contribute to personalized screening strategies.

Moreover, due to the absence of specific screening data in the Ghanaian settings, we employed simulation parameters from previous studies [7, 44, 47], as presented in Table 2. We estimated the remaining parameters using our empirical data (Table 1), with all parameters defined in Eqs (1)–(12).

### Simulation procedure

**Fig 1:**
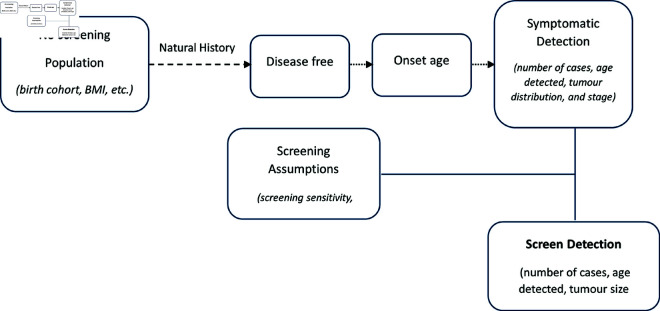
Framework of the simulation model.

The simulation study focused on the natural history of breast cancer, examining both the absence and presence of screening using specific model parameters [46]. Our simulation comprises three main components: demographics, the natural history of breast cancer in a disease population, and the screening population (See Fig 1). The demographic component covers the birth cohort of each individual, alongside their individual BMI. This was done for both models as detailed below.

#### Procedure for natural history model in the absence of screening.

1. **Simulate disease population**: We simulated 5000 births into the disease population by yearly cohort for 35 (1985-2020) different cohorts. This was done by generating a sequence of births from year 1 (birth year of the screening start age) to year 2 (birth year of the ending age) of the screening programs and replicating it 5000 times. We generated the population with the assumption that each individual has a certain probability of getting the disease at a point in time, but only a single tumor in their lifetime.
2. **Simulate tumor onset age,** *t*: For us to simulate the natural history in the absence of screening, we first simulated age at disease onset, using the probability of disease onset as presented in Eq (1), given the estimates: $A=-0.0722,\;B=1.18*10^{\left(-3\right)},\;\delta =0.0952,\;t=c(0,38:92)$ [47]. We then sampled time or age at onset using *sample* in “R" and setting *x* = *t*, *n*(size) = number of birth cohorts (e.g., 1985-2020) and probability = probability of disease onset [46].
3. **Simulate BMI-dependent inverse growth rate, *R* = *r***: We simulated BMI-dependent gamma distribution tumor growth rate *r*, based on estimates of the gamma regression function parameters in Eq (4), $\lambda_{0}$ = -0.015, $\lambda_{1}$ = 0.009, and ϕ = 1.355 [42], BMI for the simulation was sampled from a normal distribution for different population assumptions using the size of the screening population (*n*), mean and the standard deviation (mean = 25.00 , sd = 3.0). Specifically, individual BMI category populations were sampled using mean and standard deviation: (15.0, 0.50), (21.5, 0.50), (28.5, 0.50), and (45, 2.50) for underweight, normal weight, overweight, and obese, receptively.
4. **Simulate tumor volume at symptomatic,** *V*(*U*): We simulated tumor volume at symptomatic detection using Eq (5.1). Given the estimate of hazard rate η = *exp*(-9.644), tumor growth rate *r* Eq (3), volume at onset $\nu_{o}=0.065\;mm^{3}$ (given that tumor diameter at onset is 0.5 mm), and *u* was sampled from a uniform distribution *U*[0,1].
5. **Simulate size at symptomatic detection,** *d*(*U*): We simulated the size at symptomatic detection (diameter) by converting the volume, at symptomatic detection using Eq (5.2).
6. **Simulate age at symptomatic detection,** *U*: Symptomatic detection ages were simulated by adding the age at disease onset, *T*, and time to symptomatic detection $U^{\prime }$ in Eq (5.3).

### Procedure for natural history model in the presence of screening

1. **Simulate natural history without screening:** We conditioned screening assumptions on the natural history in the absence of screening.
2. **Simulate screening attendance probability**: We simulated the screening attendance probabilities. For instance, we assumed an absolute probability of all individuals attending a screening at time *t* to be perfect screening. While for imperfect screening, we assumed 0.8 of the proportion of individuals attend screening with 0.90 probability of attending a screening at time, *t*, and 0.2 proportion of individuals attend screening with 0.15 probability of attending screening at time, *t*, and 0.0 probability for not attending any screening at any time, *t* (no screening) [44, 46].
3. **Simulate tumor volume at screen detection,** *V*(*t*): The tumor volume at screen detection was simulated using Eq (2). The variables were age or time at detection *x*, the simulated age at onset *t* , growth rate, *r* (6), and initial tumor volume, $\nu_{o}$, using tumor diameter at onset of 0.5 mm, $\nu_{o}=0.065\;{\text{mm}}^{3}$.
4. **Simulate the size at detection:** *d*(*x*): We calculated the tumor size or diameter at screen detection by converting the volume to tumor size or diameter at screen as expressed in Eq (6.1).
5. **Simulate screening sensitivity, *P***_***d*(*x*)**_: We simulated screening sensitivity (see Eq (6)) for a given screening planned time or age, *x*, generated from an appropriate Bernoulli distributions. Screening sensitivity was calculated using the estimates $\beta_{0}$ = -5.04, $\beta_{1}$ = 0.56 [44]and tumor size at screen, *d*(*x*). To demonstrate sensitivity effect on high BMI under different screening interval strategies, we further simulated the screening program under different strategies using moderate sensitivity, $\beta_{0}$ = -5.04, and $\beta_{1}$ = 0.56 for underweight, normal weight ad overweight, and high sensitivity $\beta_{0}$ = -4.67 and $\beta_{1}$ = 0.65 [44] for obese.

### Screening data analysis for the association between BMI categories and screening program outcome

The effectiveness of a screening program is measured by the reduction in cancer-related death caused by the intervention, the quality of life added to the patient’s life, and the balance between benefits and harms. Common harms associated with mammography screening programs are false positives and/or overdiagnoses, which are associated with screen-detected cancers (i.e., cancer detected at a screening schedule, such as annual, biennial, or triennial). Also, false negative, which results mainly in interval-detected cancers (i.e., cancers diagnosed between scheduled screenings). In the current study, we were interested in the effect or etiological role of BMI levels (categorical) on the outcome of the screening program (detected by screening or interval) using survival analysis.

**Fig 2:**
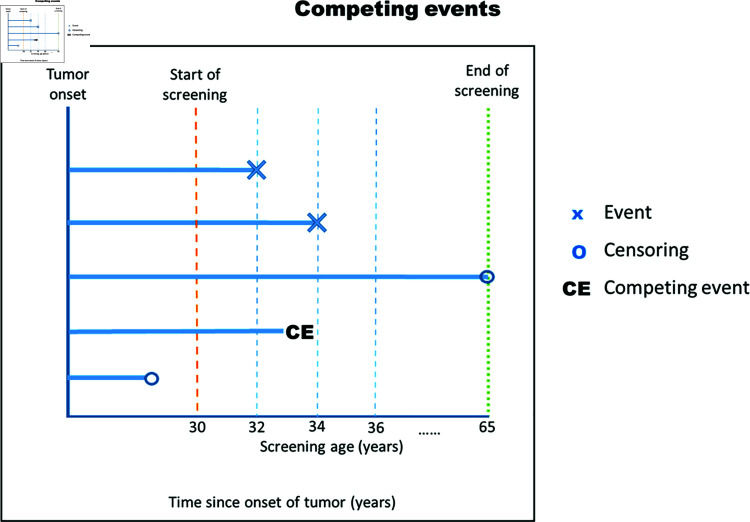
Time-to-evert study framework.

The survival regression model for this study was in the presence of competing risk, due to competing outcomes of a screening program (See Fig 2). We examined the relationship between BMI levels and screen-detected and interval-detected cancers based on the time since the onset of the disease using the cause-specific Cox proportional hazard (CPH) model [56, 57]. This model is appropriate for estimating the effect of covariate (BMI categories) on cause-specific hazard or the rate of occurrence of the event in the population who has not experienced any of the events [57, 58]. However, intuitively, survival analysis can also contribute to the clinical prognosis [57], which is very crucial in cancer research; therefore, we have partly accounted for the effect of the covariate on the absolute risk of the outcome or probability of occurrence over time, using the Fine-Gray (FGR) model that generates the subdistribution risk ratio (SHR) [56, 57]. However, due to longer FGR run time for larger data, 50000 data points were sampled from the simulated data for the SHR (S3 Table).

#### Procedure.

1. **Identify the study population (data of interest)**: The data of interest was the screened population or data.
2. **Define the follow-up date**: Follow-up begins from the date of tumor onset to detection.
3. **Define censoring times**: The censoring times were from the date of onset of the tumor to the end of the screening at the age of 65 years.
4. **Define events**: We defined two events for the screening program, the competing and primary events were cancer detected in intervals and cancer detected at screen, respectively.
5. **Indicate competing risk**: These two events were competing risks; therefore, an individual experiencing a competing event has no risk of developing the event of interest. That is the procurance of the competing event preclude the procurance of the primary event of interest
6. **Define censoring**; Censoring consists of symptomatic cases before the screening start time (loss to follow-up), and individuals with tumor onset, but the tumor was not detected by the screen or symptomatic throughout the screening period (end of follow-up). However, the latter censoring applies to the screening data.
7. **Code data for analysis**

$\text{Status}=\left\{ \begin{array}{c} 1\;-\;\text{event (screen-detected)}\\ 2\;-\;\text{competing event (interval-detected)}\\ 0\;-\;\text{censoring (Tumor not detected at screen or interval)} \end{array}\right.$

$\text{If event 1}=\left\{ \begin{array}{c} 1\;-\;\text{event 1}\\ 0\;-\;\text{censoring + event 2} \end{array}\right.$

$\text{If event 2}=\left\{ \begin{array}{c} 1\;-\;\text{event 2}\\ 0\;-\;\text{censoring + event 1} \end{array}\right.$

In the analysis, the time-to-event variable was the time since tumor onset. To account for potential confounding factors, we adjusted our model for the age of detection. The unadjusted model is given by

$$h(t|X)=h_{0}(t)\cdot exp(\tau X),$$ (7)

where h(t|X) is the expected hazard at time *t*, *h*_0_(*t*) is the baseline hazard, *X* is BMI (categorical), and τ is the coefficient of BMI. The age-adjusted model is expressed as

$$h(t|X)=h_{0}(t)\cdot exp(\tau_{1}X_{1}+\tau_{2}X_{2})$$ (8)

where $\tau_{1}$ and $\tau_{2}$ are the coefficients, and *X*_1_ and *X*_2_ are BMI (categorical) and age at detection or diagnosis, respectively.

## Results

### Maximum likelihood estimation

Table 3 presents the maximum likelihood estimates for the parameters $\lambda_{0}$, $\lambda_{1}$ and ϕ. The confidence intervals for these estimates encompass the value of 1, except ϕ. This finding suggests that the BMI covariate may not have significantly influenced the variability in breast cancer growth rates; however, it cannot be concluded that there is no impact [59]. It is important to note that with a larger sample size, there is a promising potential to demonstrate a significant association between BMI and tumor growth [42]. Notably, the direction of the effect is consistent with expectations of previous studies [42], and supports existing scientific evidence indicating that BMI may play a role in enhancing symptomatic detection. This could reflect in the breast tumor characteristics within our simulated screening program, especially because of the larger sample size. Future research should consider including additional covariates related to tumor growth, such as total breast area (TBA) and estrogen levels.

### Screening interval strategy outcome in relation to BMI categories

The study analyzed the outcomes of screening interval strategies based on BMI levels. Unlike the survival analysis, this analysis focused on descriptive analysis of the screening strategies simulated. The analysis was grouped into underweight, normal weight, overweight, and obese, respectively (Table 4). The results indicated that annual screening had a higher proportion of tumor detection than biennial and triennial screening intervals across all BMI categories. Additionally, annual screening resulted in the fewest interval cases but the highest percentage of overdiagnosis. In contrast, the biennial screening interval balanced the risks of overdiagnosis and interval cancers while achieving a reasonable detection rate. Although triennial screening had lower detection rates and less overdiagnosis, it was associated with more interval cancers. Therefore, a biennial screening interval is the most suitable option for populations across all BMI levels.

**Table 4:** Effect of different intervals on personalized screening outcome under moderate sensitivity.

Screening intervals	Sensitivity	No screening	Screening	% Change	%O’diag.	% Screen detected	% Interval cases
BMI: < 18.5 kg/m^2^							
Annual	Moderate	20460	22284	8.91	8.19	49.30	20.91
Biennial	Moderate	20460	21690	6.01	5.67	39.09	29.24
Triennial	Moderate	20460	21253	3.88	3.73	32.10	34.85
BMI: 18.5 - 24.9 kg/m^2^							
Annual	Moderate	20361	22180	8.93	8.20	48.40	21.52
Biennial	Moderate	20361	21594	6.06	5.71	38.26	29.81
Triennial	Moderate	20361	21164	3.94	3.79	31.49	35.23
BMI: 25.0 - 29.9 kg/m^2^							
Annual	Moderate	20278	22082	8.90	8.17	47.26	22.24
Biennial	Moderate	20278	21498	6.01	5.67	37.30	30.36
Triennial	Moderate	20278	21065	3.88	3.74	30.64	35.66
BMI: ≥ 30 kg/m^2^							
Annual	Moderate	20038	21850	9.04	8.29	44.89	23.86
Biennial	Moderate	20038	21252	6.06	5.71	35.24	31.64
Triennial	Moderate	20038	20835	3.98	3.82	28.77	36.79

Moreover, the findings indicated that the detection rate for cases identified through screening decreased as BMI categories increased across all three analyzed screening intervals. Specifically, the detection rates for screen-detected cases decreased from 49.30%, 39.09%, and 32.10% for underweight individuals to 44.89%, 35.24%, and 28.77% for obese individuals in the context of annual, biennial, and triennial screening intervals, respectively (see Table 4). In contrast, the detection rate for interval-detected cases increased with higher BMI across the annual, biennial, and triennial screening strategies. To address the discrepancy in the proportion of screen-detected cases between obese individuals and those of normal weight, individuals in the underweight, normal weight, and overweight categories were screened with moderate sensitivity. In contrast, the obese category was screened with high sensitivity. This adjustment improved the screening performance for obese individuals, achieving an increase of 0.78% (see S4 Table) in detection rates for annual screening compared to the moderate sensitivity approach (Table 4). However, raising the sensitivity slightly increased the proportion of overdiagnosis in the obese category.

Ultimately, the analysis highlights that the lower BMI categories respond more favorably to the sensitivity of the screening test compared to higher BMI categories [60, 61]. The study suggests that screening programs could be tailored to screen underweight individuals with lower sensitivity, those of normal and overweight status with moderate sensitivity, and obese individuals with high sensitivity to optimize overall screening performance.

### Optimal screening program in relation to BMI categories

Table 5 presents a descriptive analysis of the optimal screening program while considering individual BMI risk. The simulated BMI risk-based screening program assumed varying attendance rates and sensitivity levels based on BMI categories. Screening was conducted for individuals aged 30 to 65 every two years, with moderate sensitivity for underweight, normal weight, and overweight categories and high sensitivity for the obese category. The mean age of the population not detected during screening was 47 years, while the mean age of those detected by screening was 46 years.

**Table 5:** Optimal screening program in relation to BMI category.

Summary	No screening	Screening
Mean Age	47	46
1st Quantile Age	39	38
Median Age	47	46
3rd Quantile Age	54	53
Number Diagnosed	20318	22129
Tumor size 0-9 mm	610 (3.00 %)	6385 (28.85%)
Tumor size 10-19 mm	3074 (15.23%)	7811 (35.30%)
Tumor size 20-50 mm	10843 (53.37%)	4132 (18.67%)
Tumor size ≥ 50 mm	5791 (28.50%)	3800 (17.17%)

In terms of tumor size distribution for interval-detected tumors, the majority were between 20 and 50 mm (53.37%), with a significant portion exceeding 50 mm (28.50%), indicating a high median tumor size distribution among symptomatic cases. Conversely, the program detected many small tumors (64.15%) measuring 20 mm or less, accompanied by a notable overdiagnosis rate of 8.18% for cases detected through screening.

### Breast tumor characteristics

#### Tumor size distribution from the simulation.

**Fig 3:**
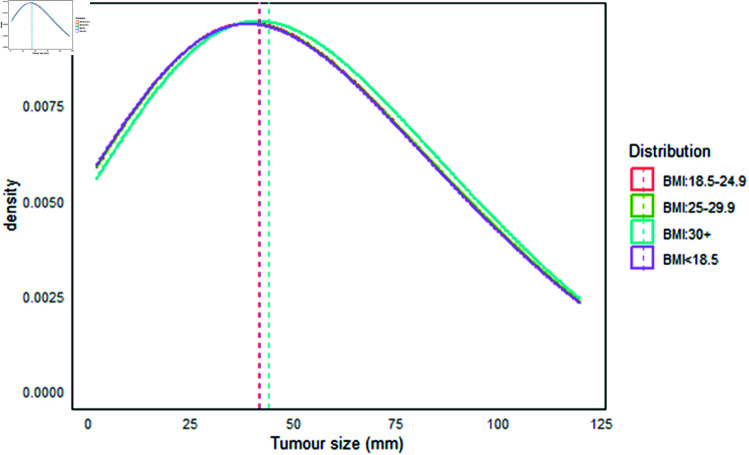
Tumor size distribution (mm) of interval-detected cancers.

**Fig 4:**
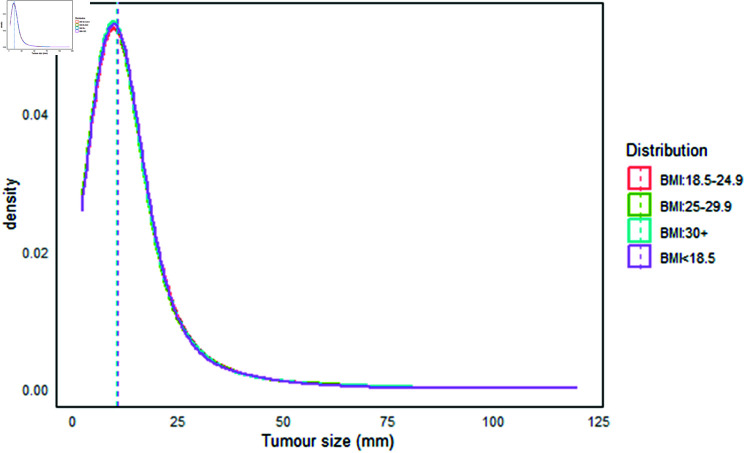
Tumor size distribution (mm) of screen-detected cancers.

In Fig 3, the overall median tumor size for interval cancers (with a maximum tumor size of 120 mm) was 42 mm, with a mean of 50 mm (SD: ±30 mm) and an interquartile range (IQR) of 24-69 mm. In contrast, in Fig 4 the overall median tumor size for screen-detected tumors was 10 mm, with a mean of 12 mm (SD: ±10 mm) and an IQR of 7-15 mm. When examining tumor sizes for interval cancers across different BMI levels, the median sizes were 41.86 mm, 42.03 mm, 42.18 mm, and 44.22 mm for BMI categories of <18.5, 18.5-24.9, 25.0-29.9, and ≥ 30kg/m^2^, respectively. For screen-detected cancers, the median sizes at the same BMI levels were 10.65 mm, 10.48 mm, 10.52 mm, and 10.58 mm.

Our findings indicate that higher body size is associated with larger tumor sizes in interval cases [48]. However, the sizes were nearly consistent across all four BMI categories for screen-detected tumors. This suggests that screening programs effectively reduce the risk of larger tumor sizes, particularly in obese women, corroborating the idea that obese individuals appear to be associated with smaller tumors at preclinical or at early stage [62]. Our results also align with an earlier study [63], which reported that introducing screening mammography has increased the detection of smaller tumors.

#### Growth rates in relation to detection mode.

**Fig 5:**
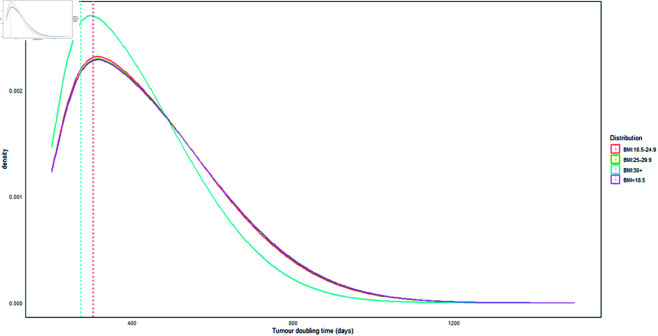
Tumor doubling time (days) of interval-detected cancers.

**Fig 6:**
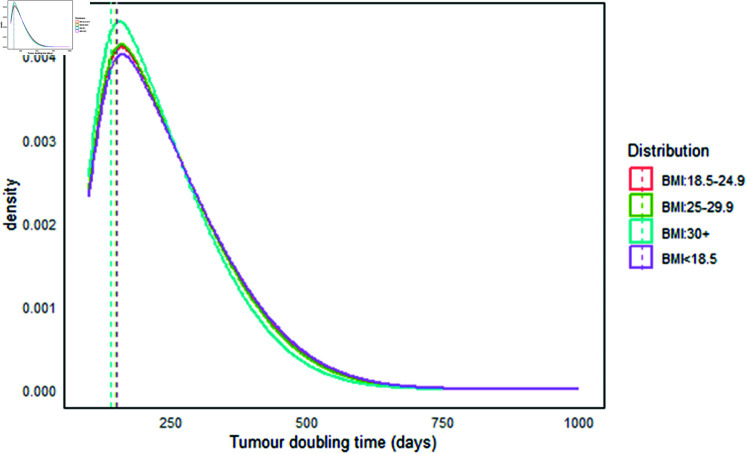
Tumor doubling time (days) of screen-detected cancers.

In this study, tumor growth rate was measured as the volume doubling time of the tumor. The overall median doubling time (DT) was 150 days, with a mean of 174 days (SD: ±127 days) and IQR of 71 to 251 days. For interval cancers, the median tumor doubling times based on different BMI levels were as follows: 152 days for BMI < 18.5, 150 days for BMI 18.5-24.9, 149 days for BMI 25.0-29.9, and 140 days for BMI ≥ 30 kg/m^2^ (see Fig 5 and Table 6). In contrast, the median tumor doubling times for screen-detected cancers were 302 days for BMI < 18.5, 304 days for BMI 18.5-24.9, 303 days for BMI 25.0-29.9, and 272 days for BMI ≥ 30 kg/m^2^ (see Fig 6 and Table 6). The findings indicated that tumor doubling times were shorter for obese women in both interval-detected and screen-detected cancers. This suggests that individuals with higher body weight experience faster tumor growth compared to those with lower body weight [64]. The clinical implication is that women with a higher BMI are likely to benefit more from a frequent screening program than those with a lower BMI. This approach allows for the detection of small tumors before they double in size and become symptomatic [65], potentially leading to improved patient outcomes. Therefore, screening individuals at intervals based on their BMI category may offer more clinical advantages than a “one-size-fits-all” approach, which applies a single screening interval for everyone regardless of BMI levels.

**Table 6:** Tumor doubling time in relation to BMI categories.

Detection mode	No. of cases	Mean ± (SD)	1Q	Median	3Q
**BMI < 18.5 kg/m^2^**					
Screen-detected	8677	330 (± 220)	151	302	375
Interval-detected	2638	177 (± 130)	73	152	256
**BMI 18.5-25.0 kg/m^2^**					
Screen-detected	273544	333 (± 222)	152	304	479
Interval-detected	81056	174 (± 127)	71	150	252
**BMI 25.0-29.9 kg/m^2^**					
Screen-detected	248763	332 (± 221)	152	303	478
Interval-detected	74971	172 (± 127)	71	149	250
**BMI ≥30 kg/m^2^**					
Screen-detected	25978	296 (± 196)	137	272	425
Interval-detected	10440	164 (± 120)	67	140	237

1Q and 3Q are first and third quartiles respectively.

#### Tumor presence time in relation to interval cancers.

**Fig 7:**
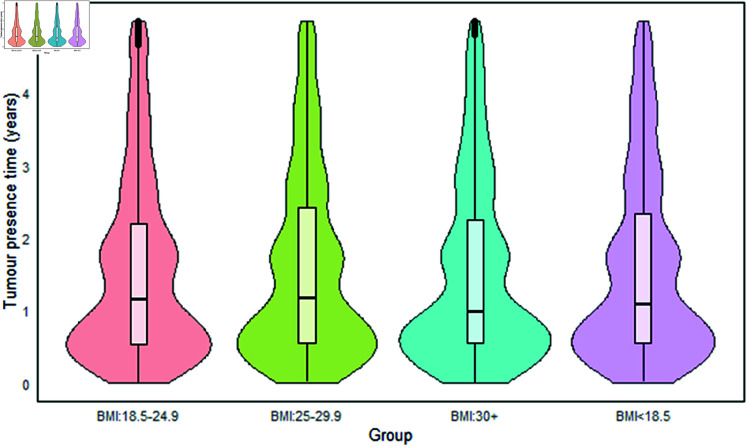
Tumor presence time (years) for interval cancers.

In the present study, the preclinical phase (mean sojourn time) is referred to as the tumor presence time, which is the duration a tumor takes from onset before it is detected symptomatically [66]. In Fig 7, the median duration of tumor presence time for interval-detected cancers is 2.13 years, with a mean of 5.08 (SD: ±7.0) and IQR of 0.74 to 6.88 years. The median presence times for interval cancers at different BMI levels are as follows: 2.11 years (mean: 5.04, SD: ±6.63, IQR: 0.76 to 6.72) for BMI < 18.5, 2.17 years (mean: 5.11, SD: ±6.69, IQR: 0.75 to 6.93) for BMI 18.5 to 24.9, 2.13 years (mean: 5.07, SD: ±6.69, IQR: 0.74 to 6.85) for BMI 25.0 to 29.9, and 1.93 years (mean: 4.69, SD: ±6.36, IQR: 0.70 to 6.23) for BMI ≥ 30 kg/m^2^. This suggests that healthier women, or those with normal body weights, tend to have a longer preclinical phase compared to obese women or those with excess body weight. This observation explains the higher sensitivity and increased detection rates during the preclinical phase for individuals with normal body weight, as shown in Table 6 [67]. It indicates that individuals with a normal BMI have a greater window of opportunity for a screening program to detect small tumors before they become clinically evident. This is in contrast to those with a higher BMI, who have a shorter preclinical phase due to a more rapid tumor doubling time. As a result, individuals with higher BMI may require shorter screening intervals, the periods between each screening, to improve the chances of detecting tumors at the preclinical stage.

The results regarding tumor characteristics indicate that individuals with higher body weight generally have a shorter tumor presence duration than those with lower body weight, mainly due to a faster doubling time [64]. This suggests that individuals within the normal BMI range have a longer preclinical phase, during which small tumors can be detected. Consequently, they are likely to benefit more from the standard biennial (24-month) screening intervals than obese individuals (see Association between BMI categories and screening outcomes). Our findings imply that obese individuals could benefit from more frequent screenings to improve the early detection of tumors before clinical symptoms manifest, given their faster tumor doubling time [65]. This indicates that tumor growth may not be uniform for each individual; instead, it may vary based on body weight. Therefore, the risk of interval cancer could also differ according to body weight distribution at the time of screening. As a result, screening intervals should be tailored to individuals based on their body weight. This personalized approach could lead to earlier detection and improved outcomes, providing reassurance about the potential impact of the research on patient care. Specifically, a potential screening interval of 14 to 18 months may be more suitable for obese individuals.

### Association between BMI categories and screening outcomes

To further demonstrate our findings, we analyzed the relationship between BMI categories and screening outcomes (including screen-detected and interval-detected cancers) using survival analysis on the preclinical time-to-event data from the simulated screening program. Table 7 presents the cause-specific hazard ratio (HR) by BMI category (we assumed a maximum tumor size of 50 mm for the screening program). For screen-detected cancers (primary event), the HR for normal weight was 1.140 (95%CI: 1.115-1.165), indicating a 14% higher risk of screen-detected cases than the reference group. Conversely, the HR for interval-detected cancers (competing event) was 0.942 (95%CI: 0.912-0.973), suggesting a 5.8% reduction in the risk of interval-detected cases for the normal weight group compared to the reference group. Additionally, the HR for overweight individuals was 1.132 (95%CI: 1.108-1.158) for screen-detected cases, representing a 13.2% higher risk of screen-detected cases for the overweight group compared to the reference group. Similarly, the HR for overweight individuals under interval-detected cases was 0.960 (95%CI: 0.929-0.992), indicating a 4% reduction in the risk for this group compared to the reference group.

**Table 7:** Cause-specific hazard ratio of screen and interval cancers by BMI category.

BMI category (kg/m^2^)	N o. of cases	Unadjusted HR (95% CI)	Age-adjusted HR (95% CI)
**Screen-detected cancers**			
Underweight (<18.5)	8097	Ref	Ref
Normal Weight (18.5–24.9)	272340	1.152 (1.127-1.178)	1.140 (1.115-1.165)
Overweight (25.0–29.9)	246434	1.145 (1.120-1.170)	1.132 (1.108-1.158)
Obese (≥30)	26523	1.149 (1.120-1.178)	1.129 (1.102-1.158)
**Interval-detected cancers**			
Underweight (<18.5)	3738	Ref	Ref
Normal Weight (18.5–24.9)	104384	0.930 (0.900-0.960)	0.942 (0.912-0.973)
Overweight (25.0–29.9)	96647	0.947 (0.917-0.979)	0.960 (0.929-0.992)
Obese (≥30)	11986	1.077 (1.038-1.117)	1.099 (1.060-1.140)

Finally, the HR for obese individuals is 1.129 (95%CI: 1.102-1.158) for screen-detected cancers, which indicates a 12.9% higher risk compared to the reference group. Similarly, the HR for obese individuals under interval-detected cases is 1.099 (95%CI: 1.060-1.140), suggesting a 0.9% higher risk of interval-detected cases for the obese group compared to the reference group. Since the HR CIs did not include 1, this finding is statistically significant and indicates an increased risk despite the values being close to 1. In summary, the obese group is slightly associated with a higher risk of breast cancer detection, regardless of the detection mode [68].

The findings indicate that obese individuals have a slightly higher risk of interval-detected cancers than normal body weight individuals compared to screen-detected cancers. This aligns partly with previous research on the association between BMI and the prevalence of interval-detected cancers [69], however, it may not necessarily align with all risk prediction studies including [70], and [71] because they explain binary outcomes for mammography which may be impacted by screening sensitivity features such as breast density, while our study focused on time-to-even to contribute to mammography outcome driven by tumor-related growth factors which are critical for screening interval design, however our findings agrees with previous studies [69–71] emphasis on poorer breast cancer prognosis in obese women.

Furthermore, survival analysis was performed in the presence of competing risks; therefore, we generated a cumulative incidence curve using the cumulative incidence function (CIF) instead of the regular Kaplan–Meier (KM) method, based on the violation of the KM assumption (non-informative censoring) [72] which leads to an overestimation of the probability of events [72]. The cumulative probability of detecting cancer at screen 50 years after tumor onset was 0.684 for underweight individuals, 0.723 for normal-weight individuals, 0.718 for overweight individuals, and 0.689 for obese individuals. Similarly, the cumulative probability of detecting cancer symptomatically in the presence of screening (interval-detected cancers) 50 years after tumor onset was 0.316 for underweight individuals, 0.277 for normal-weight individuals, 0.282 for overweight individuals, and 0.311 for obese individuals.

**Fig 8:**
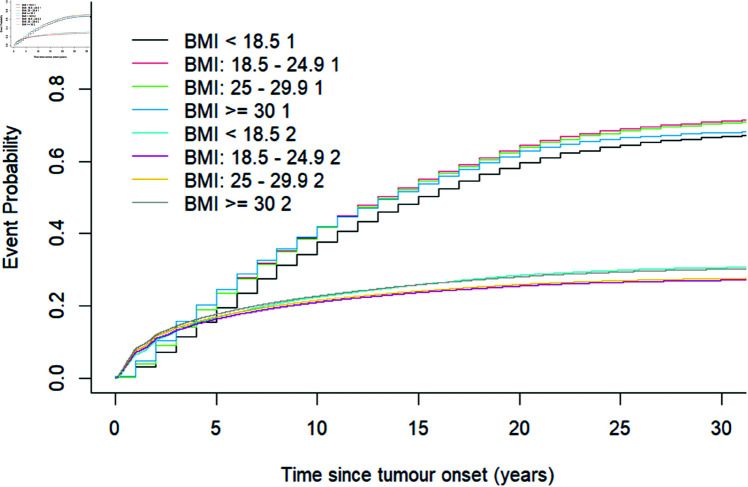
Cumulative incidence for screen (labeled 1 after each BMI category) and interval (labeled 2 after each BMI category) detected cancers by BMI categories from onset of the tumor to detection in the presence of screening.

The $\chi^{2}$ statistic for difference in cumulative incidence for the screen-detected cancer (Event 1) was 5.22$\times 10^{6}$ with a p-value of 0 (p-value < 0.05) on 3 degrees of freedom. Similarly, the $\chi^{2}$ statistic for difference in cumulative incidence for the interval-detected cancer (Event 2) was 2.49$\times 10^{2}$ with a p-value of 0 (p-value < 0.05) on 3 degrees of freedom. This suggests a significant difference in cumulative incidences for BMI categories or groups for screen and interval-detected cancers, respectively. Additionally, the high $\chi^{2}$ statistic for screen-detected incidences (Event 1) further suggests a high cumulative probability and high significance in cumulative incidences for screen-detected than for interval-detected incidences (Event 2) as illustrated in Fig 8.

Therefore, the study showed that the BMI categories significantly impact screening outcomes or time to detection. The apparent discrepancy between the non-significant association between BMI and tumor growth rate and the significant association between BMI categories and screening outcomes may be attributed to two factors. First, in the tumor growth estimate, we were interested in the continuous BMI variable to account for the complexity of continuous biological tumor growth distribution and variability, which was crucial to effectively model the natural history and elucidate tumor characteristics. In contrast, the analysis of the association between BMI and screening outcome focused on categorical BMI variables, which examine the frequency and types of outcomes, to help understand patterns in screening data. Second, the sample size for tumor growth rate estimation was limited; it is possible that a larger sample size, like the simulated screening data used for the analysis of the association between BMI and screening outcome, would yield different results. This study enhances the concept of “personalized screening” as our results highlight that BMI categories influence screening outcomes. It advocates for varying sensitivity levels and screening frequencies according to an individual’s BMI category and other risk factors. This targeted approach aims to reduce the risk of interval-detected cancers while also minimizing the potential for overdiagnosis and other harms linked to screening.

## Discussion

This study used a tumor growth model incorporating BMI and a simulation approach to examine various strategies for determining mammography screening intervals. Our goal was to identify the optimal screening strategy for different BMI levels and to explore the relationship between BMI and screening outcomes. This research is vital as it contributes to personalized screening by recommending specific schedules tailored to various BMI categories. Such an approach aims to minimize the risk of overdiagnosis and interval cancers while increasing the chances of detecting smaller tumors [11]. Our findings could guide population-based breast cancer screening programs in Ghana, guiding policies that ensure an effective program with fewer interval-related cancers and a higher detection rate of smaller tumors.

The study results showed a larger tumor size for higher BMI in populations not engaged in screening. Previous studies similarly argued that obese women who do not undergo screening or have interval-detected cancers are likely to present with larger tumors [29, 73]. This is due to tumor-promoting factors that are typically low in women with lower body weight [29]. Conversely, when screening is available, smaller tumors are detected. This point is further supported by [29], which recommends that mammography screening programs can identify smaller tumors among obese women, highlighting the potential benefits of population-based screening, particularly for this demographic. These findings align with prior research [42]. However, the inability of our study to address the finding in [42]—which indicated that high BMI and larger breast size jointly delay interval cancers—was due to our focus on the independent association of BMI with tumor growth and characteristics [73], compounded by the lack of breast size data in our analysis.

Moreover, we observed that obese women experience shorter tumor doubling times, indicating a faster growth rate for breast tumors in this group compared to women of lower weight [64]. The results also demonstrated that obese women have a shorter tumor presence time compared to those with lower body weight. This suggests that the interval between tumor onset and clinical manifestation is shorter for women with higher BMI. Quantifying this preclinical phase is vital for determining appropriate screening intervals [41].

Additionally, we established an association between BMI and screening program outcomes using Cox proportional hazard models to estimate hazard ratios across various BMI categories despite the non-significant effect of BMI on continuous tumor growth. The difference between the non-significant association of BMI with tumor growth rate and the significant association between BMI categories and screening outcomes may be due to two key factors. First, the tumor growth model employed a continuous BMI variable to better capture the complexity of tumor growth, while the analysis of screening outcomes utilized categorical BMI variables to examine patterns in the results. Second, the sample size for estimating tumor growth was limited, whereas the simulated screening data had a larger sample size, which may have contributed to the differing results.

Our findings indicated that obese women are more likely to experience interval-detected cancers rather than screen-detected cancers. In contrast, women with normal body weight are more inclined to have screen-detected cancers, likely due to their longer tumor presence time compared to their obese counterparts. Prior research also indicated a slight association between high BMI and the prevalence of interval-detected cancers versus screen-detected cancers [69], corroborating our results. This further supports earlier findings that link higher BMI with larger tumor sizes, as most interval cases tend to present with larger tumors. Ultimately, our findings suggest that obese women may have poorer prognoses compared to other BMI categories, underscoring the need for tailored screening intervals based on body weight [41]. The results from the cumulative incidence function model, which analyzed time-to-event plots for different BMI levels, found that women with normal body weights had a higher probability of screen-detected event occurrence compared to those who were obese.

Although in this study, we utilized the carcinogenesis risk model based on the Moolgavkar-Venzon-Knudson framework adopted by Strandberg *et al*. [45] to estimate the probability of disease onset in populations lacking specific screening data, such as Ghana, by adjusting the onset evaluation for the Ghanaian population using comparative GLOBOCAN age-standardized data for West Africa (Ghana) and Northern Europe (Sweden) [7]. The results were consistent with the demographics of the Ghanaian or West African populations (See tumor detection age distribution in Tables 1 and 5), and a sensitivity analysis with a ±10% variation showed no significant sensitivity effects (See S1 Table). However, despite this practical adjustment to calibrate the onset to the Ghanaian context, the model (Eq 1) relies on parameter estimates from [47], which could introduce limitations to our study due to genetic variations between the two regions. Furthermore, in this study, we introduced a novel aspect to our research by modeling the BMI-dependent tumor growth rate (Eq 4) and age at symptomatic detection (Eq 5) specifically for Ghana. These estimates, derived from our empirical data, were consistent with previous studies [42, 45]. However, it is essential to note that our empirical data were derived from invasive tumors in clinical patients, which may affect our tumor growth rate estimates. Additionally, the data collection occurred at a single, prominent health facility, potentially skewing patient demographics due to socioeconomic factors. As a result, our findings may not accurately reflect the Ghanaian population. Therefore, our results should be regarded as a guide rather than a foundation for establishing population-based screening policies for Ghana or other low- and middle-income countries. The assessment of personalized screening programs focused solely on the BMI risk factor, as there is insufficient information on other risk factors. This limited focus may overlook the complexities related to breast cancer risk. Future research should incorporate additional risk factors, such as family history, hormone replacement therapy, and menopausal status, to improve risk stratification and develop more targeted screening strategies.

We also simulated our screening program, leveraging screening test sensitivity parameters from Andersson *et al*. [44] to model screening sensitivity (Eq 6). This was calibrated for the Ghanaian population by comparing detection proportions [51] and conducting sensitivity analyses with a ±10% variation (S2 Table). This approach proved beneficial, as the detection outcomes mirrored proportions found in previous studies and countries with established screening programs [44, 46]. The sensitivity analysis indicated that the parameters did not significantly respond to variation. However, this approach may still influence our estimates and introduce uncertainties, as the calibration process relies on assumptions that might not fully encompass the genetic and demographic complexities of the Ghanaian population. Ultimately, our study did not demonstrate a significant impact of BMI on tumor growth. However, the direction of the parameters was consistent with prior studies’ findings [42].

Despite its noted limitations, this study contributes to the limited research on continuous growth models examining the relationship between BMI levels and tumor growth. Its potential impact on public health policies is significant. By gaining stakeholder support for a pilot screening program, we could enhance prospective breast cancer data collection. This could lead to more accurate estimation of model parameters for the Ghanaian population, inform the development of population-based screening policies, and provide clinicians with valuable insights into the disease’s natural history. Such insights could be instrumental in tailoring treatment protocols effectively, thereby improving patient outcomes.

## Conclusion

The study’s findings highlight the potential for improved screening practices to make a significant difference in cancer outcomes. The association between BMI categories and outcomes from population-based screening is a key discovery. The observation that biennial screening is the most beneficial approach for all BMI levels, surpassing annual and triennial strategies, contributes to similar recommendations for enhanced cancer screening. Women with a high BMI are linked to larger interval-detected tumor sizes, shorter tumor doubling times (indicating a faster tumor growth rate), and shorter durations of tumor presence compared to women with normal body weight. This underscores the importance of screening obese women at shorter intervals than their normal-weight counterparts, a strategy that can help reduce the occurrence of interval-related cancers in population-based screening programs. These insights into improved screening practices will undoubtedly inspire and motivate efforts to enhance breast cancer treatment and management, as interval cancers tend to have higher proliferation rates, may spread more quickly to other parts of the body, and are associated with lower survival rates.

## Supporting information

S1 Table Sensitivity analysis of onset risk. (PDF)

S2 Table Sensitivity analysis of screening test sensitivity. (PDF)

S3 Table Comparing hazard ratios of different models. (PDF)

S4 Table Effect of different screening interval strategies outcome under different sensitivity. (PDF)
