# Supplementary material for: Optimizing breast cancer screening strategies for women with different BMI levels in Ghana: A simulation-based study on BMI-dependent tumor growth model
Source: PLOS Glob Public Health. 2025 Jul 28;5(7):e0004953. doi: 10.1371/journal.pgph.0004953 (PMC12303353; doi:10.1371/journal.pgph.0004953)
Supplement: S1 Table — (PDF) [file pgph.0004953.s001.pdf]

Supporting information:

S1 Table: Sensitivity analysis of onset risk

	**Optimal**		**Risk increased by 10%**		**Risk decreased by 10%**	
Summary	No screening	Screening	No screening	Screening	No screening	Screening
Mean Age	47	46	47	47	47	46
1st Quantile Age	39	38	40	38	39	38
Median Age	47	46	48	47	47	46
3rd Quantile Age	55	54	55	54	54	54
Number Diagnosed	20867	21915	20759	21942	21096	21917
Tumour size 0-9 mm	3.16%	20.09%	3.49%	21.74%	2.66%	17.29%
Tumour size 10-19 mm	16.40%	35.63%	17.61%	37.16%	14.33%	33.17%
Tumour size 20-50 mm	58.25%	29.15%	59.53%	28.45%	56.24%	30.30%
Tumour size > 50 mm	22.19%	15.13%	19.37%	12.64%	26.75%	19.24%
