# Supplementary material for: Optimizing breast cancer screening strategies for women with different BMI levels in Ghana: A simulation-based study on BMI-dependent tumor growth model
Source: PLOS Glob Public Health. 2025 Jul 28;5(7):e0004953. doi: 10.1371/journal.pgph.0004953 (PMC12303353; doi:10.1371/journal.pgph.0004953)
Supplement: S2 Table — (PDF) [file pgph.0004953.s002.pdf]

Supporting information:

S2 Table: Sensitivity analysis of screening test sensitivity

	Optimal		**Sensitivity increased by 10%**		**Sensitivity decreased by 10%**	
**Summary**	**No screening**	**Screening**	**No screening**	**Screening**	**No screening**	**Screening**
Mean Age	47	46	47	46	47	46
1st Quantile Age	39	38	39	38	39	38
Median Age	47	46	47	46	47	46
3rd Quantile Age	55	54	56	55	55	54
Number Diagnosed	20867	21915	20879	21988	20850	21822
Tumor size 0-9 mm	3.16%	20.09%	3.15%	21.58%	3.18%	18.61%
Tumor size 10-19 mm	16.40%	35.63%	16.50%	35.78%	16.43%	34.50%
Tumor size 20-50 mm	58.25%	29.15%	58.24%	37.73%	58.08%	31.06%
Tumor size $>$ 50 mm	22.19%	15.13%	22.12%	14.91%	22.31%	15.82%
