# Supplementary material for: Optimizing breast cancer screening strategies for women with different BMI levels in Ghana: A simulation-based study on BMI-dependent tumor growth model
Source: PLOS Glob Public Health. 2025 Jul 28;5(7):e0004953. doi: 10.1371/journal.pgph.0004953 (PMC12303353; doi:10.1371/journal.pgph.0004953)
Supplement: S3 Table — (PDF) [file pgph.0004953.s003.pdf]

Supporting information:

S3 Table: Comparing hazard ratios of different models

Variable	Subdistribution Hazard Ratio		Cause-Specific Hazard Ratio	
**Unadjusted**				
	**Screen-detected**	**Interval-detected**	**Screen-detected**	**Interval-detected**
BMI $\leq$ 18.5	Ref	Ref	Ref	Ref
BMI:18.5-24.9	1.22 (1.12-1.33)	0.796 (0.706-0.897)	1.152 (1.127 -1.178)	0.9296 (0.8998-0.9604)
BMI:25 - 29.9	1.20 (1.10-1.31)	0.812 (0.720-0.916)	1.145 (1.120-1.170)	0.9473 (0.9168-0.9788)
BMI $\geq$ 30	1.17 (1.07-1.29)	0.877 (0.765- 1.005)	1.149 (1.120-1.178)	1.0768 (1.0380-1.1171)
**Adjusted for age at detection**				
	**Screen-detected**	**Interval-detected**	**Screen-detected**	**Interval-detected**
BMI $\leq$ 18.5	Ref	Ref	Ref	Ref
BMI:18.5-24.9	1.213 (1.114-1.320)	0.804 (0.714-0.907)	1.1398 (1.1149 -1.1653)	0.9420 (0.9118-0.9733)
BMI:25-29.9	1.197 (1.099-1.302)	0.821 (0.728-0.926)	1.1324 (1.1076-1.1577)	0.9603 (0.9294-0.9922)
BMI $\geq$ 30	1.167 (1.061-1.284)	0.895 (0.781-1.025)	1.1293 (1.1016-1.1578)	1.0993 (1.0596-1.1404)

Total no. of observation= 770448
Total number of events = 553394
Total number of competing risk = 216755
Total number of censoring = 299
