# Supplementary material for: Optimizing breast cancer screening strategies for women with different BMI levels in Ghana: A simulation-based study on BMI-dependent tumor growth model
Source: PLOS Glob Public Health. 2025 Jul 28;5(7):e0004953. doi: 10.1371/journal.pgph.0004953 (PMC12303353; doi:10.1371/journal.pgph.0004953)
Supplement: S4 Table — (PDF) [file pgph.0004953.s004.pdf]

Supporting information:

S4 Table: Effect of different screening interval strategies outcome under different sensitivity

Screening intervals	Sensitivity	No screening	Screening	% Change	%O'diag.	% Screen detected	% Interval cases
			BMI: < 18.5 kg/m^2				
Annual	Moderate	20460	22284	8.91	8.19	49.30	20.91
Biennial	Moderate	20460	21690	6.01	5.67	39.09	29.24
Triennial	Moderate	20460	21253	3.88	3.73	21.00	34.85
			BMI: 18.5 - 24.9 kg/m^2				
Annual	Moderate	20361	22180	8.93	8.20	48.40	21.52
Biennial	Moderate	20361	21594	6.06	5.71	38.26	29.81
Triennial	Moderate	20361	21164	3.94	3.79	31.49	35.23
			BMI: 25.0 - 29.9 kg/m^2				
Annual	Moderate	20278	22082	8.90	8.17	47.26	22.24
Biennial	Moderate	20278	21498	6.01	5.67	37.30	30.36
Triennial	Moderate	20278	21065	3.88	3.74	30.64	35.66
			BMI: ≥ 30 kg/m^2				
Annual	High	20046	22234	10.91	9.84	48.05	22.04
Biennial	High	20046	21637	7.94	7.35	39.04	29.16
Triennial	High	20046	21210	5.81	5.48	32.76	34.09
